# Regulation of Signal Transduction and Role of Platelets in Liver Regeneration

**DOI:** 10.1155/2012/542479

**Published:** 2012-07-03

**Authors:** Takeshi Nowatari, Kiyoshi Fukunaga, Nobuhiro Ohkohchi

**Affiliations:** Department of Surgery, Graduate School of Comprehensive Human Sciences, University of Tsukuba, Tsukuba 305-8575, Japan

## Abstract

Among all organs, the liver has a unique regeneration capability after sustaining injury or the loss of tissue that occurs mainly due to mitosis in the hepatocytes that are quiescent under normal conditions. Liver regeneration is induced through a cascade of various cytokines and growth factors, such as, tumor necrosis factor alpha, interleukin-6, hepatocyte growth factor, and insulin-like growth factor, which activate nuclear factor **κ**B, signal transducer and activator of transcription 3, and phosphatidyl inositol 3-kinase signaling pathways. We previously reported that platelets can play important roles in liver regeneration through a direct effect on hepatocytes and collaborative effects with the nonparenchymal cells of the liver, including Kupffer cells and liver sinusoidal endothelial cells, which participate in liver regeneration through the production of various growth factors and cytokines. In this paper, the roles of platelets and nonparenchymal cells in liver regeneration, including the associated cytokines, growth factors, and signaling pathways, are described.

## 1. Introduction

Liver regeneration is a physiopathological phenomenon of quantitative recovery from the loss of liver mass to compensate for decreased hepatic volume and impaired function [1, 2]. Although numerous studies have shown that a variety of genes, cytokines, growth factors, and cells are involved in liver regeneration, the exact mechanism of regeneration and the interaction between hepatocytes and cytokines are not fully understood [3]. Liver regeneration is a critical issue related to clinical morbidity and mortality in drug-induced liver injury and after surgery including hepatectomy or living-donor liver transplantation [4, 5]. The temporal development of the signaling pathways specifically activated during liver regeneration may be described in three phases: a priming phase, involving the transition of quiescent hepatocytes from G0 into the G1 phase of the cell cycle; a proliferation phase during which the progression of the entire hepatocyte population occurs; a termination phase during which cell proliferation is suppressed and regeneration is terminated at a defined point [6, 7]. Hepatocytes are not terminally differentiated; rather, the cells are in proliferative quiescence (the G0 phase) but can rapidly enter a cell division cycle upon stimulation [6]. The cytokines/signaling pathways and proteins that are important during the priming phase of regeneration include tumor necrosis factor alpha (TNF*α*)/nuclear factor *κ*B (NF*κ*B), interleukin-6 (IL-6)/signal transducer and activator of transcription 3 (STAT3), activator protein-1 (AP-1), and mitogen-activated protein kinase (MAPK)/extracellular signal-regulated protein kinase (ERK) [8–14]. Phosphatidyl inositol 3-kinase (PI3K)/Akt is also immediately activated after hepatectomy and plays an important antiapoptotic role during liver regeneration [15]. During the proliferation phase, hepatocytes express various cell cycle proteins that guide the replication process, including hepatocyte growth factor (HGF) and epidermal growth factor (EGF) [16, 17]. Essentially, the major factors involved in the termination phase comprise transforming growth factor beta (TGF*β*) and activins [6].

Seventy percent of the cell number or 80% of the liver volume is composed of hepatocytes, and the remaining cells consist of nonparenchymal cells, including Kupffer cells, liver sinusoidal endothelial cells (LSECs), hepatic stellate cells, and lymphocytes, which are thought to play an important role in cytokine release [18]. Upon activation, Kupffer cells are reported to produce both inflammatory cytokines, such as, TNF*α* and IL-6, and such growth factors as insulin-like growth factor (IGF)-1 [19]. LSECs have also been reported to produce IL-6 and HGF after hepatectomy and activated hepatic stellate cells mainly produce HGF [20, 21].

Platelets play pivotal roles in thrombosis and hemostasis, but an increasing variety of extrahemostatic functions of platelets have been recognized [22]. Platelets contain many growth factors, such as, platelet-derived growth factor (PDGF), vascular endothelial growth factor (VEGF), HGF, IGF, EGF and TGF*β*, and some cytokines [23–29]. Furthermore, platelets play certain roles of stimulation or acceleration during hepatocyte proliferation [30]. We recently reported that platelets play a very important role in liver regeneration after hepatectomy and that HGF and IGF-1 derived from platelets are essential for hepatocyte proliferation [23, 31–33]. Platelets play a direct role in hepatocytes during liver regeneration and also a cooperative role with nonparenchymal cells in the liver [33, 34]. Kupffer cells contribute to the accumulation of platelets in the liver, which can subsequently induce liver regeneration [31, 33, 34]. Furthermore, the direct contact between platelets and LSECs induces sphingosine 1-phosphate (S1P) release from the platelets, which subsequently induces the secretion of IL-6 from LSECs. The LSEC-derived IL-6 promotes DNA synthesis in hepatocytes via the STAT3 pathway [35].

Herein, we describe the cellular and molecular mechanisms of liver regeneration and the functions of some critical signaling pathways involved in hepatocyte proliferation. In particular, we focus on the roles of platelets in liver regeneration.

## 2. Cytokines, Growth Factors, and Signaling Pathways in Liver Regeneration

**Figure 1:**
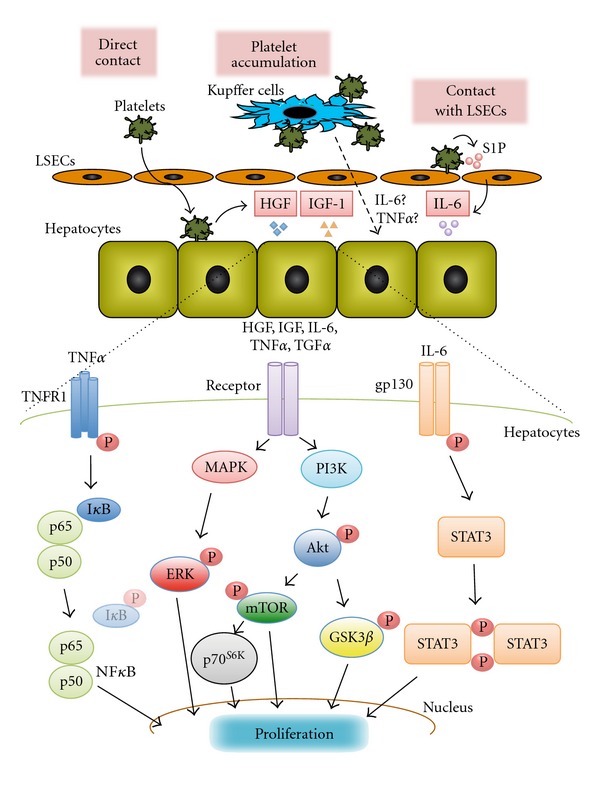
*The Roles of Platelets and the Signal Transductions Identified as the Major Cascades in Hepatocyte Proliferation.* After liver injury, Kupffer cells can play a crucial role in the accumulation of platelets in the liver sinusoids and the production of cytokines, such as, interleukin-6 (IL-6) and tumor necrosis factor alpha (TNF*α*). Moreover, Kupffer cells induce the translocation of platelets into the space of Disse and the direct contact between platelets and hepatocytes, which trigger the release of growth factors, such as, hepatocyte growth factor (HGF) and insulin-like growth factor (IGF)-1, which are necessary for hepatocyte proliferation. Platelets also have direct contact with liver sinusoidal endothelial cells (LSECs), which trigger the release of sphingosine 1-phosphate (S1P). S1P induces the secretion of IL-6 from LSECs, which promotes hepatocyte proliferation. During hepatocyte proliferation, IL-6 binding induces both the dimerization and the phosphorylation of gp130 and promotes the docking site of signal transducer and activator of transcription 3 (STAT3). STAT3 is then phosphorylated and translocated to the nucleus. Phosphatidyl inositol 3-kinase (PI3K)/Akt signaling pathway is essential for the platelet-induced hepatocyte proliferation and is activated via the receptor tyrosine kinases through the stimulation of HGF, IGF, IL-6, and many other signaling molecules. Phosphorylated Akt activates glycogen synthase kinase 3*β* (GSK3*β*), which induces DNA synthesis and cellular mitosis in hepatocytes. Other downstream Akt factors including mTOR and p70^S6K^ also play critical roles in regulating the growth of hepatocytes. Mitogen-activated protein kinase (MAPK)/extracellular signal-regulated protein kinase (ERK) signaling pathway is also immediately activated after hepatectomy and thereafter phosphorylated ERK translocates to the nucleus. TNF*α*/nuclear factor *κ*B (NF*κ*B) signaling pathway is activated via the TNF receptor 1 (TNFR1). NF*κ*B is a heterodimer composed of two subunits, p65 and p50, with inactivated by inhibitor of NF*κ*B (I*κ*B). After stimulation with TNF*α*, NF*κ*B is activated by the removal of I*κ*B from its p65 subunit, followed by the migration to the cell nucleus.

Liver regeneration occurs through the proliferation of all of the existing mature cellular populations including the hepatocytes, biliary epithelial cells, LSECs, Kupffer cells, and hepatic stellate cells. All of the cells proliferate to rebuild the lost hepatic tissue, and the hepatocytes are known to be the first cells to proliferate [36]. Hepatocytes exhibit a mitogenic response to various growth factors and cytokines, such as, HGF, IGF-1, IL-6, TNF*α*, EGF, TGF*β*, and PDGF [1, 23]. These growth factors and cytokines lead to the subsequent activation of downstream transcription cascades, which effect the transition of the quiescent hepatocytes into the cell cycle and progression beyond the restriction point in the G1 phase [1]. The cascades also result in the activation of transcription factors and signal transduction pathways, such as, NF*κ*B, STAT3, MAPK/ERK, PI3K/Akt, AP-1, and CCAAT/enhancer-binding protein-*β*, which subsequently induce hepatocyte proliferation [8–15, 37–41]. Among these transcription factors and corresponding signal transductions, the TNF*α*/NF*κ*B, IL-6/STAT3, PI3K/Akt, and MAPK/ERK pathways are identified as the major cascades during the process of liver regeneration (Figure 1).

### 2.1. TNF*α*/NF*κ*B Signaling Pathway

TNF*α* is a crucial cytokine during the priming phase of liver regeneration and activates the NF*κ*B transcription factor via the TNF receptor 1 (TNFR1) in hepatocytes [42]. After partial hepatectomy, the production of TNF*α* is upregulated mainly in Kupffer cells; NF*κ*B is activated within 30 minutes after partial hepatectomy, and the activation usually lasts no longer than 4-5 hours [1, 43]. During hepatocyte proliferation, NF*κ*B is a heterodimer composed of two subunits, p65 and p50, which are assembled in the cytosol; the complex is inactivated by inhibitor of NF*κ*B (I*κ*B), which binds to the p65 subunit. After stimulation with TNF*α*, NF*κ*B is activated by the removal of I*κ*B from its p65 subunit; the activated NF*κ*B then migrates to the cell nucleus, which regulates the G0/G1-to-S phase transition [44–46]. It was reported that the administration of a TNF*α* antibody or the knockout of TNFR1 in mice results in delayed liver regeneration after partial hepatectomy [8, 47]. In Kupffer cell-depleted mice, liver regeneration was impaired because of the loss of TNF*α* and NF*κ*B [48].

### 2.2. IL-6/STAT3 Signaling Pathway 

The STAT3 pathway, which is activated by such cytokines as IL-6, is known to play a crucial role in cell proliferation [49, 50]. Kupffer cells express TNFR1 on their surface and activate themselves in an autocrine fashion. Because the promoter region of the IL-6 gene contains the NF*κ*B-binding site, IL-6 can also be produced by the activated Kupffer cells [51]. Recently, both LSECs and Kupffer cells were reported to produce IL-6 after hepatectomy [20, 33]. IL-6 binding causes the dimerization of gp130, which is the ubiquitously expressed signaling receptor molecule for the IL-6 family, and the activation of the intracellular tyrosine kinase that phosphorylates gp130 and creates the docking site of STAT3 [52]. STAT3 is then phosphorylated and translocates to the nucleus. STAT3 is activated slower than NF*κ*B, becoming detectable at 1 to 2 hours after partial hepatectomy and lasting approximately 4–6 hours [1]. It was reported that hepatocytic mitosis in STAT3-knockout mice was significantly suppressed during liver regeneration after partial hepatectomy [53].

### 2.3. PI3K/Akt Signaling Pathway 

The PI3K/Akt pathway has been known as a survival pathway functioning in antiapoptosis [54–56]. Recently, it was revealed that the PI3K/Akt pathway is responsible for regulating cell growth and determining cell size and functions [57–62]. In addition, Akt and downstream signals cause the compensatory hypertrophy of hepatocytes when cell proliferation is impaired [49]. The pathway is initiated by the activation of receptor tyrosine kinases or G protein-coupled receptors by HGF, IL-6, TNF*α*, TGF*α*, and many other signaling molecules [63–67]. HGF is a potent growth factor that is mainly derived from LSECs and activated HSCs and promotes proliferation and DNA synthesis in hepatocytes in a paracrine fashion [20, 68]. C-met is a tyrosine kinase receptor on the surface of hepatocytes that binds to HGF, and HGF/c-met signaling activates PI3K, which then recruits Akt to the site of membranes and subsequently phosphorylates Akt [65, 69, 70]. Phosphorylated Akt activates glycogen synthase kinase 3*β* (GSK3*β*), which induces DNA synthesis and cellular mitosis in hepatocytes [49, 71]. Furthermore, other downstream Akt factors, such as, mTOR and p70^S6K^, play critical roles in liver regeneration by regulating cell growth in addition to activated GSK3*β* [49, 60, 61]. It was reported that the specific PI3K inhibitor LY294002 abolished DNA synthesis in growth factor-stimulated hepatocytes [72].

### 2.4. MAPK/ERK Signaling Pathway 

Once the MAPK/ERK pathway has been activated, ERK translocates to the nucleus where it can regulate the transcriptional activity of many immediate early genes [73]. The signaling cascade is activated by growth factors, such as, IGF-1 and EGF and is involved in the regulation of G1 phase progression during liver regeneration *in vivo *and in hepatocyte proliferation *in vitro *[13, 74–76]. In particular, IGF-1 signals activate both the PI3K/Akt and MAPK/ERK pathways, leading to the control of genes involved in hepatocyte proliferation and in protecting against apoptotic cell death [77].

## 3. Roles of Platelets in Liver Regeneration

It is known that platelets release local mediators and interact with leukocytes and endothelial cells to modulate inflammatory responses [78]. Platelets are involved in wound healing and tissue repair in addition to hemostasis and inflammation and can be associated with liver regeneration or tissue repair in liver injury. Platelets contain both the proteins needed for hemostasis and also many growth factors, such as, PDGF, HGF, IGF, VEGF, EGF, and TGF*β*, which are required for tissue regeneration [23–29, 79–81]. In addition, platelets also contain certain cytokines, serotonin and lipid mediators, such as, S1P, ADP, and ATP [79, 82–84]. We previously reported on the relationship between platelets, nonparenchymal cells and hepatocytes during liver regeneration [23, 31, 33, 35] (Figure 1).

### 3.1. Direct Effect on Hepatocytes

We previously reported that platelets have a potent role in promoting liver regeneration after partial hepatectomy in mice by activating the Akt and ERK signaling pathways and stimulating hepatocyte proliferation *in vitro* [23, 31]. It is clear that the number of platelets affects liver regeneration during the priming phase after hepatectomy [31, 33, 85, 86]. As observed by transmission electron microscopy, some platelets were found to translocate into Disse's spaces through the fenestration of LSECs and had direct contact with hepatocytes [31], which is similar to wound healing in which platelets become activated and release essential growth factors and cytokines by contact with collagen and other extracellular matrixes [79]. We previously reported that, when platelets and hepatocytes were separated by a permeable membrane, the platelets had no proliferative effect on the hepatocytes, suggesting that the direct contact between the cells was essential for inducing hepatocyte proliferation [23]. Growth factors in platelets, such as, IGF-1 and HGF, activated the Akt and ERK1/2 pathways and caused a proliferative effect on hepatocytes [23]. As it was reported that human platelets do not contain a significant amount of HGF, IGF-1 is the most important mediator for liver regeneration in humans [23, 87]. Platelets did not exert a proliferative effect on hepatocytes in the presence of LY294002, which inhibits the activation of the Akt pathway, suggesting that the Akt pathway is an important signal and that the activators of the Akt pathway are key molecules involved in the direct proliferative effect by platelets [23]. Furthermore, even under conditions of Kupffer cell depletion, platelets had a strong effect in hepatocyte proliferation through the phosphorylation of Akt, suggesting that Akt activation could compensate for liver regeneration when the TNF*α*/NF*κ*B pathway derived from Kupffer cells was impaired [33].

### 3.2. Relationship between Platelets and Nonparenchymal Cells 

Nonparenchymal cells in the liver, including Kupffer cells, LSECs, and hepatic stellate cells, are involved in the process of liver regeneration [36]. Because Kupffer cells are close to hepatocytes, the release of these mediators after partial hepatectomy may initiate the liver regeneration process [8, 88, 89]. Kupffer cells produce inflammatory cytokines, such as, TNF*α* and IL-6, and such growth factors as IGF-1 are generally presumed to be an important source of hepatic TNF*α*, which is a key component in the process of hepatocyte proliferation during liver regeneration [42, 90]. The depletion of Kupffer cells was reported to result in delayed liver regeneration because of the loss of TNF*α* and NF*κ*B and the decrease of IGF-1 [33, 48, 91]. LSECs, which comprise 70% of the sinusoidal cells in the liver, are known to produce immunoregulatory and proinflammatory cytokines, including HGF, IL-6, interleukin-1, and interferon [20, 92, 93]. Under conditions of Kupffer cell depletion, the phosphorylation of STAT3 was detected in the regenerating liver after hepatectomy, suggesting that IL-6 could be produced by LSECs (instead of Kupffer cells) at a sufficient level to activate STAT3 [33]. We previously reported that platelets could function in collaboration with nonparenchymal cells during liver regeneration [33–35].

#### 3.2.1. Relationship between Platelets and Kupffer Cells

Kupffer cells can be associated with the accumulation of platelets in the liver, which induces liver regeneration [33, 34]. It was reported that platelet accumulation in the liver after hepatectomy or other types of liver injury, such as, ischemic reperfusion or lipopolysaccharide administration, depended to some extent on Kupffer cells [31, 34, 94, 95]. Platelets in the liver sinusoids were mostly surrounded by the well-developed cell processes of Kupffer cells without phagocytosis [34]. Furthermore, the depletion of Kupffer cells resulted in the abolition of the accumulation and the migration of platelets in the liver [33, 34]. These results indicated that the cellular interactions between platelets and Kupffer cells play important roles in platelet behavior in the liver. In thrombocytosis, more platelets were recruited into the liver, providing high levels of IGF-1 and HGF, which induced the subsequent activation of downstream signal transductions, such as, the PI3K/Akt and MAPK/ERK pathways and advanced hepatocyte mitosis [33].

#### 3.2.2. Relationship between Platelets and LSECs

We previously reported that platelets induced hepatocyte proliferation through LSEC activation [35]. Direct contact between platelets and LSECs triggered the secretion of S1P from the platelets, which induced the secretion of IL-6 from the LSECs; thereafter, the increase of IL-6 caused the activation of the STAT3 pathway in hepatocytes, Akt and ERK1/2 activation and the promotion of hepatocyte proliferation [35]. In addition, platelets caused the proliferation of LSECs and induced the secretion VEGF and IL-6 from the LSECs by activating the Akt and ERK1/2 pathways [35].

## 4. Conclusion

The liver is a vital organ in which the mechanisms of regeneration are orchestrated by a complex network of cytokines and growth factors. Nonparenchymal cells in the liver, such as, Kupffer cells, LSECs, and hepatic stellate cells, participate in liver regeneration with respect to both their own proliferation and effects on hepatocyte proliferation [36]. In particular, the Kupffer cells and LSECs produce various growth factors and cytokines that are involved in liver regeneration [19, 20]. Platelets contain various types of growth factors and cytokines and comprise another important factor involved in liver regeneration [23, 31]. In summary, platelets have a direct effect on stimulating hepatocyte proliferation and cooperative with Kupffer cells and LSECs during liver regeneration.
